# From Sensors to Systems: Real-Time Human Performance and Health Monitoring

**DOI:** 10.3390/s26144608

**Published:** 2026-07-21

**Authors:** Borja Muniz-Pardos, Elena Comadran de Barnola, Yannis P. Pitsiladis

**Affiliations:** 1 EXER-GENUD (Growth, Exercise, NUtrition and Development) Research Group (S72_23R), International Federation of Sports Medicine (FIMS) Collaborating Center of Sports Medicine, University of Zaragoza, 50009 Zaragoza, Spain; bmuniz@unizar.es (B.M.-P.); elena.comadran.de.barnola@euncet.es (E.C.d.B.); 2 Centre for Exercise Science and Medicine (CESAME), Hong Kong Baptist University, Hong Kong SAR, China; 3 Department of Biology, Hong Kong Baptist University, Hong Kong SAR, China; 4 Department of Sport and Health Sciences, Hong Kong Baptist University, Hong Kong SAR, China

**Keywords:** real-time monitoring, wearable sensors, physiological sensing, core temperature, artificial intelligence, exertional heat illness, sensor ecosystems, environmental sensing, human performance

## Abstract

**Highlights:**

**What are the main findings?**

- Real-time human performance monitoring should move beyond isolated wearable devices toward integrated physiological sensor ecosystems that combine thermal, cardiovascular, biomechanical, environmental and contextual data.
- Sensor accuracy alone is insufficient for high-stakes decision-making; physiological sensing systems require time-synchronised data streams, multi-level validation, uncertainty-aware modelling and fit-for-purpose interpretation.

**What are the implications of the main findings?**

- AI-assisted decision support may improve pacing, cooling, medical review, withdrawal and return-to-play decisions, but only if models are transparent, calibrated, prospectively validated and robust to individual and contextual variability.
- Future sensor ecosystems should embed data governance, interoperability, athlete autonomy, regulatory compatibility and human oversight to avoid false reassurance, surveillance, bias and misuse of physiological data.

**Abstract:**

Wearable and environmental sensors are increasingly used to monitor human performance and health in real time, but their practical value remains limited when signals are interpreted in isolation or through context-independent algorithms. This Perspective focuses on real-time physiological monitoring in elite sport, using exertional heat illness risk and physiological strain as a high-stakes worked example. Our work further illustrates how such monitoring could move from device-level measurement toward fit-for-purpose sensor ecosystems. We argue that such ecosystems should be defined not by multimodal data collection alone, but by five core pillars: time-synchronised sensing, context-specific interpretation, multi-level validation, uncertainty-aware modelling and embedded governance—each evaluated against a defined high-stakes decision rather than device accuracy alone. Accurate decision-making in this context may require integration of thermoregulatory, cardiovascular, biomechanical and environmental data streams. Emerging artificial intelligence approaches may support prediction and decision support, but only if models are transparent, calibrated, prospectively validated and robust to individual and contextual variability. We also examine unresolved risks, including algorithmic bias, false reassurance, athlete surveillance, proprietary opacity, data asymmetry and regulatory constraints. While the framework and evidence presented here are anchored in elite sport, we discuss where its core validation and governance principles may extend to clinical, occupational and military settings, and where the underlying evidence remains sport-specific. The proposed framework is intended to complement, rather than replace, existing digital health, athlete management and cyber–physical systems by specifying the validation and governance requirements needed for high-stakes human performance monitoring.

## 1. Introduction: From Fragmented Monitoring to Physiological Sensor Ecosystems

Real-time monitoring of human performance and health is entering a critical transition. Over the past decade, wearable devices, environmental sensors and digital platforms have made it increasingly possible to capture, analyse and transmit physiological, biomechanical and environmental signals during training, competition and occupational tasks [1,2]. Yet the field remains largely fragmented. Many systems still rely on isolated metrics, proprietary algorithms or context-independent outputs that are interpreted without sufficient evidence of reliability, sensitivity, validity or contextual applicability [3,4]. This creates a fundamental limitation: data availability has increased, but biological interpretability and decision utility have not advanced at the same pace.

The central challenge is therefore no longer simply whether a sensor can measure a signal, but whether that signal can be meaningfully integrated into a validated system capable of representing the physiological state of an individual in context. In elite-sport, military, occupational and clinical environments, decisions may depend on subtle interactions between thermal strain, cardiovascular load, mechanical workload, environmental exposure, behavioural responses and individual susceptibility. No single wearable signal can adequately capture this complexity. Human performance and health monitoring must therefore evolve from isolated devices toward integrated physiological sensor ecosystems.

Exertional heat illness (EHI) provides a useful example of this broader problem. EHI remains a major and preventable threat to athlete health, particularly in endurance and intermittent high-intensity sports performed under hot or humid conditions [5]. A systematic review of organised sport reported EHI incidence rates ranging from 0.01 to 4.19 cases per 1000 athlete-exposures, and from 0.01 to 54.54 cases per 100 participants, with the highest participant-based rates observed in endurance-type events such as running, cycling and adventure racing [5]. In international athletics championships, heat-related illnesses have been reported at an average incidence of 11.7 cases per 1000 registered athletes, with rates increasing as apparent temperature rises [6]. Accordingly, recent consensus recommendations from the International Olympic Committee emphasise the need for heat-specific planning, environmental monitoring, cooling strategies and sport-specific regulations to protect athletes during events held in hot conditions [7].

Environmental indices such as wet-bulb globe temperature (WBGT) remain essential for population-level risk stratification, but they cannot quantify the physiological strain experienced by an individual athlete [8]. Equivalent WBGT values may correspond to different heat-loss potentials depending on whether conditions are hot-dry or warm-wet, further limiting the capacity of a single environmental index to represent individual heat strain [9]. Moreover, environmental exposure is spatially heterogeneous. Recent in-stadium monitoring studies have shown that heat exposure may vary substantially within the same sporting venue, with temperature differences of up to 10 °C between locations [10]. Similarly, field campaigns during endurance events have demonstrated that official meteorological stations may fail to capture the microclimatic conditions actually experienced by athletes along a route [11]. These observations support the need to combine environmental sensing with individual physiological monitoring.

The inclusion of biomechanical metrics derived from inertial measurement units (IMUs), instrumented insoles, GPS or power meters further extends the capacity to interpret athlete–environment interactions. When combined with environmental sensing (through geospatial and mobile meteorological stations), this allows for a more accurate analysis of the athlete–environment interaction, closing the loop between internal strain and external load. This multi-layered approach is fundamental to the emerging field of sensor-based human performance science, particularly when data may inform high-stakes decisions such as pacing modification, cooling, medical evaluation or athlete withdrawal.

A central challenge in real-time human performance monitoring is construct validity: determining whether the measured signal truly represents the physiological construct of interest [3,12]. Core temperature, skin temperature, heart rate, movement acceleration, power output and environmental heat stress are often interpreted as indicators of physiological strain, yet each reflects only one component of a complex, regulated system. Therefore, the validity of a sensor ecosystem should not be judged solely by the accuracy of individual devices, but by the ability of the integrated system to represent the underlying physiological state and support meaningful decisions.

**Figure 1:**
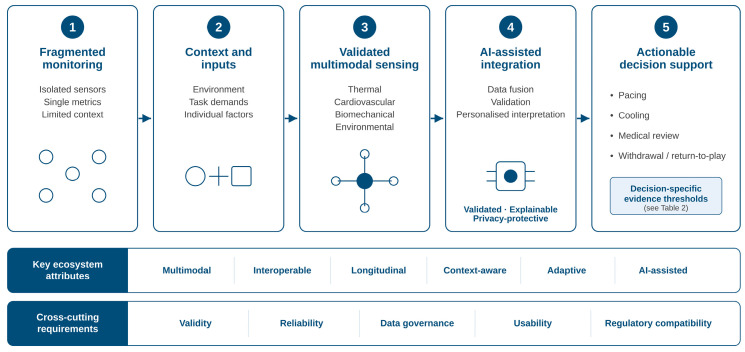
Physiological sensor ecosystem for real-time human performance and health monitoring.

In this Perspective, we define a physiological sensor ecosystem as an integrated monitoring architecture that combines multiple validated data streams into a context-aware and actionable representation of human physiological state. Given the breadth of potential applications, we deliberately anchor the Perspective in a single high-stakes worked example—exertional heat illness and thermal strain in sport—while keeping the framework general enough to transfer to clinical, occupational and military settings. The scope is therefore conceptual rather than device-specific: we do not aim to validate a particular sensor or algorithm, but to define the validation and governance requirements that any ecosystem should meet before informing a high-stakes decision. The primary objective of this Perspective is therefore to characterise this validation-and-governance architecture in the sport setting, where exertional heat illness offers the most developed body of applied evidence available to us. References elsewhere in this manuscript to clinical, occupational and military applications are illustrative of where the same underlying logic may reasonably apply. Such ecosystems should be multimodal, interoperable, longitudinal, context-aware, adaptive and AI-assisted, extending recent calls for scientifically validated, ethically governed and collaborative technology innovation in elite sport [1,2]. Figure 1 summarises this proposed ecosystem framework, and Table 1 sets it against related digital-health, cyber–physical, athlete-management, digital-twin and multimodal-biosensing frameworks. The specific contribution of this Perspective is two-fold. First, we define a hierarchical, decision-anchored validation scheme (Table 2) in which a sensing system is judged not by device accuracy alone but by its fitness for a named high-stakes decision (e.g., athlete withdrawal). Second, we embed governance and regulatory-compatibility requirements specific to elite-sport and extreme-environment settings, where data collection may be coercive and feedback may be restricted in competition. To our knowledge, no existing digital-health, athlete-management or cyber–physical framework combines these two elements for this decision context. Other elements are context-dependent and optional—including digital-twin simulation, edge or federated computation, and the specific sensing modalities—and should be selected according to the target decision rather than added by default.

## 2. Sensor Accuracy and Validation: From Signal Measurement to Fit-for-Purpose Use

The integrity of physiological monitoring depends on accurate measurement rather than estimation [3,12]. Non-invasive core-temperature technologies offer major practical advantages, but most systems do not measure deep body temperature directly. Instead, they estimate it from peripheral signals such as skin temperature, heat flux and, in some cases, heart rate, often using proprietary algorithms [14,15]. This distinction is critical. A sensor-derived estimate may show acceptable group-level agreement with a reference method while still producing clinically meaningful errors in specific individuals or under specific environmental conditions [14,16]. In such settings, the key risk is not only measurement error, but erroneous physiological interpretation. A small bias may be acceptable for recreational feedback but unacceptable when the output informs cooling, pacing, medical evaluation or withdrawal decisions.

Ingestible thermistors are widely used [13,17] as a reference method for assessing gastrointestinal temperature during dynamic field exercise, particularly when continuous monitoring is required outside the laboratory. However, their interpretation requires consideration of ingestion timing, gastrointestinal transit, fluid intake and potential sensor lag [18,19,20]. Our research has benchmarked newer sensor systems against these standards. For example, during an elite triathlon race [21], an ultra-distance running challenge in extreme conditions [22], a long-distance running competition in a warm-humid environment [23], or during an expedition of mountaineers in extreme cold [24], our team demonstrated that wearable and ingestible systems can be synchronised with motion and cardiovascular data to provide a more holistic picture of physiological stress, with these data transmitted in real time.

Validation should be conceptualised as a multi-level process rather than a single comparison against a reference device. First, analytical validity determines whether the sensor accurately captures the physical signal under controlled conditions. Second, criterion validity establishes agreement with an accepted reference standard during the specific activity, posture, intensity and environmental conditions in which the device is intended to be used. Third, reliability and sensitivity to change determine whether the device can detect meaningful within-individual changes over time. Finally, ecological and decision validity determines whether the system remains accurate in real-world conditions and whether its outputs can improve health or performance decisions [25,26]. To clarify this concept, Table 2 summarises validation as a hierarchical, multi-level process extending from technical signal accuracy to real-world decision utility. For a withdrawal or medical-intervention decision, we propose that a core-temperature system should meet the agreement criteria established for core temperature—a systematic bias < 0.1 °C with 95% limits of agreement within ±0.4 °C against an ingestible thermistor during exercise in the heat [13]—together with high data completeness in the field (e.g., <10% data loss); systems that do not meet this standard should be restricted to screening or trend monitoring rather than individual withdrawal decisions. These thresholds are grounded in the core-temperature literature. Byrne and Lim [13] defined acceptable agreement between core-temperature methods as a systematic bias below 0.1 °C with 95% limits of agreement within ±0.4 °C, a criterion since used to benchmark non-invasive devices. Independent validations illustrate how demanding it is: the CORE sensor has shown biases and limits of agreement exceeding these bounds during cycling and heat exposure [14,16], with comparable variability reported for other estimated-core-temperature devices [15,27]. The ingestible-thermistor reference itself is sensitive to ingestion timing, gastrointestinal transit and fluid intake, which must be controlled for [13,17,18,19,20]. We therefore present the values in Table 2 as evidence-informed reference points rather than universal cut-offs. Crucially, these thresholds require context-specific adaptation. The acceptable margin of error should scale with the consequence of the decision—a stricter standard is warranted for individual withdrawal or medical intervention than for population-level screening or retrospective trend analysis—and may need to be re-derived for different populations, body sites, activities and environmental conditions. The values proposed here should therefore be treated as a starting point for prospective, decision- and context-specific validation.

Environmental measurement is another critical component of real-time monitoring ecosystems. Fixed and mobile meteorological stations can characterise microclimatic variability directly at the field of play, including differences related to shade, solar radiation, wind exposure, surface characteristics and spectator infrastructure. When synchronised with individual physiological data, these environmental measurements allow for a more precise interpretation of athlete–environment interactions than reliance on official meteorological stations alone. Ultimately, the goal is not to replace direct measurement with estimation, but to fuse multiple data layers—core temperature, cardiovascular strain, movement metrics, and environment—into a reliable, validated framework.

## 3. From Data to Decisions: Modelling, Integration and Uncertainty

Real-time physiological data are only as meaningful as the systems that interpret them [2]. Isolated metrics, even when measured accurately, provide limited insight. The scientific challenge—and opportunity—lies in integrating these data streams into a coherent ecosystem capable of guiding health and performance decisions. In ecosystem-based monitoring, artificial intelligence (AI) should not be viewed as an additional technological layer added onto wearable devices, but as the computational framework that enables multimodal data fusion, individualised interpretation and adaptive decision support [28].

In this context, AI should be understood as a family of modelling approaches rather than a generic technological label [28]. Supervised learning models may be used to classify risk states or estimate continuous outcomes such as core-temperature trajectory, cardiovascular drift or probability of uncompensated heat strain (Table 3). Probabilistic and Bayesian models may be useful when uncertainty is clinically relevant, because they can update risk estimates as new physiological and environmental data arrive [29]. Time-series methods, including state-space models, recurrent architectures, temporal convolutional networks or transformer-based approaches, may help capture lagged relationships between workload, environment and physiological response [30]. Digital-twin approaches could eventually support counterfactual simulation, such as estimating how a given athlete might respond to altered pacing, cooling or environmental exposure [31], but these models require substantial individual-level data and careful validation before operational use. Edge–AI approaches may reduce latency and improve data protection by processing selected signals locally, whereas federated learning may allow for model development across teams, events or institutions without centralising sensitive athlete data [32,33]. Regardless of the modelling strategy, the critical issue is not algorithmic complexity but whether the model is calibrated, interpretable, robust to missing data and valid for the intended population and decision.

**Figure 2:**
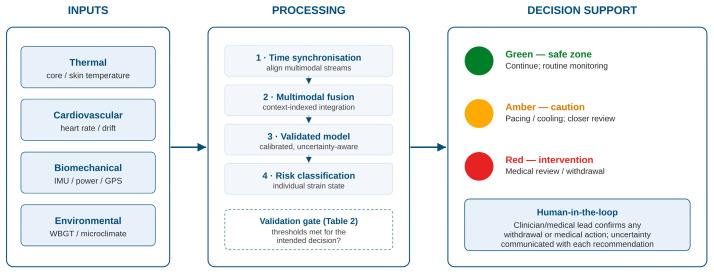
Traffic-light decision-support framework. Time-synchronised multimodal inputs (thermal, cardiovascular, biomechanical and environmental) are fused and classified by a validated, uncertainty-aware model; graded green/amber/red outputs map to routine monitoring, pacing or cooling, and medical review or withdrawal, respectively. Operational use is gated by the decision-specific thresholds in Table 2, and any withdrawal or medical action is confirmed by a human decision-maker.

One possible implementation is a traffic-light decision-support framework that integrates thermal, cardiovascular, biomechanical and environmental signals to classify individual risk states in real time (Figure 2). Such systems remain under development and require prospective validation before they can be used to guide operational or medical decisions [34]. This traffic-light system operates as follows: green indicates safe zones of thermal strain, amber signals caution, and red prompts intervention. However, designing such systems is highly complex, requiring high-frequency data, real-time analytics, and robust AI training datasets. The development of AI-assisted risk systems requires careful definition of the target label. A “red” alert may represent excessive core temperature, rapid cardiovascular drift, a predicted probability of EHI, impaired thermoregulatory compensation, or a combination of these outcomes. These constructs are related but not interchangeable. Therefore, algorithmic development should begin with explicit labelling rules, temporal alignment of multimodal data streams, handling of missing or delayed signals, and prospective validation against clinically or operationally meaningful outcomes. In high-stakes environments, model performance should not be evaluated only using global accuracy, but also sensitivity, specificity, calibration, false-alarm burden, interpretability and the consequences of missed events. Accordingly, we recommend that any operational deployment (i) pre-registers the target label and alarm thresholds, (ii) reports calibration and false-alarm burden alongside accuracy, (iii) requires prospective field validation against a clinically or operationally meaningful outcome, and (iv) retains a human decision-maker in the loop for any withdrawal or medical action.

Translating these approaches into trusted operational tools requires a staged validation-and-deployment pathway rather than direct release. A model should first run in a silent or “shadow” mode in which its outputs are recorded but not acted upon, enabling prospective analyses; progression to operational use should be contingent on pre-specified performance (calibration, sensitivity and false-alarm burden; Table 2) being demonstrated in the target population and environment. Once deployed, models require continuous monitoring for data drift and periodic recalibration, transparent documentation of inputs and failure modes, fail-safe default behaviour when signals are missing or out of range, and an auditable human-in-the-loop process for any withdrawal or medical action (Figure 2). Crucially, trust should rest on independent, external validation rather than on internal development metrics, and each recommendation should be accompanied by an explicit statement of uncertainty.

These recommendations should be read with appropriate humility. AI in physiological monitoring is a rapidly evolving field: model architectures, validation methods and regulatory expectations are all changing quickly, and no single modelling approach has yet been established as best-in-class for this use case. Model performance can also degrade over time through data drift and distribution shift, so a system that performs well today is not guaranteed to remain valid. We therefore frame the guidance above as durable principles—decision-anchored validation, calibrated uncertainty, human oversight and continuous monitoring—rather than fixed prescriptions, and we emphasise that, given this uncertainty, AI should augment rather than replace expert and clinical judgement until stronger prospective evidence is available.

A useful illustration of the ecosystem approach comes from long-term attempts to integrate real-time sensing into elite endurance sport. These programmes are presented as one illustrative line of work rather than a comprehensive survey. Since 2015, successive deployments have highlighted both the potential and the practical barriers of real-time human telemetry. Early prototypes developed within the Sub2 Marathon Project demonstrated the feasibility of athlete-centred monitoring, but also revealed that isolated devices are insufficient unless embedded within a broader sensing, analytics and decision-support architecture. It all started in 2015 with the “Kenenisa” wearable system—named after Kenenisa Bekele—which featured a prototype with a battery life of two hours and one minute, designed to match the target marathon duration. This represented an early field implementation of the Sub2 real-time monitoring system, which is further explained elsewhere [1,35]. This ecosystem-based philosophy—integrating measurement, prediction, and feedback—now defines our work. Subsequent applications in triathlon, road running, ultra-endurance events, cold-weather expeditions, combat-sport head-impact monitoring and cycling have reinforced the same principle: the scientific value of human sensing depends less on any single device than on the validity, interoperability and interpretability of the broader ecosystem in which the device operates (Figure 1).

Similar integration challenges are evident beyond the authors’ own deployments. In military settings, real-time physiological status monitoring has been explored for exertional heat illness mitigation, highlighting both the promise of continuous sensing and the difficulty of translating alerts into operational decisions [36]. Occupational heat-stress monitoring has also tested wearable systems for estimating core temperature and heat strain in workers [27], while elite and team-sport environments increasingly combine GPS, inertial sensors, heart rate, wellness and workload data within athlete-management systems [37]. These examples show that the movement toward integrated monitoring is field-wide, but also that most current systems remain limited by validation, interoperability, governance and decision-specific evidence. Beyond these examples, comparable efforts by independent groups—across military [36], occupational [38] and team-sport [39] settings—reach the same conclusion, indicating that the case for decision-anchored ecosystems does not rest on any single research programme.

## 4. Evidence Standards, Commercial Translation and Scientific Guardrails

Despite the rapid expansion of the wearable technology market, scientific validation remains limited relative to the number of devices and biometric outcomes advertised [40]. Earlier critiques suggested that only a small minority of consumer-grade technologies had undergone formal validation [40], and more recent umbrella-review evidence indicates that approximately 11% of commercially available wearable devices have been validated for at least one biometric outcome [4]. While such tools may satisfy the curiosity of recreational users, they lack the precision required for elite sport, clinical use, or military applications where decisions may have direct implications for safety, medical management or operational performance. Importantly, validation of one variable, such as heart rate or step count, cannot be extrapolated to other outputs such as energy expenditure, sleep quality, fatigue, physiological strain or injury risk. Therefore, scientific claims should be outcome-specific, context-specific and population-specific [3]. Of note, the relatively small subset of systems that truly measure physiological parameters demonstrates how high the standard is. The issue is therefore not only whether a device has been validated, but whether the specific output being marketed has been validated for the intended population, context and decision.

A further limitation is that many wearable systems are validated, marketed and interpreted as standalone devices [41]. Interoperability is therefore not merely a technical convenience, but a scientific requirement for ecosystem-level interpretation [41]. This fragmented approach impedes progress because health and performance cannot be reduced to one metric. Science demands ecosystems—networks of interoperable sensors, algorithms, and analytics that communicate and contextualise each other’s signals. Without this integration, data remain difficult to interpret or implement. A key implication is that progress should be evaluated less by competition between individual devices and more by the capacity to integrate validated signals into interoperable, decision-specific systems, bringing environmental, physiological, and biomechanical sensors into one validated, AI-assisted system. Such integration may not yet be commercially attractive, but it is the only scientifically defensible way forward.

## 5. Future Applications: From Monitoring to Context-Specific Decision Support

The future of real-time monitoring will not be defined by the number of sensors that can be attached to the body, but by the capacity to transform multimodal data into integrated biological intelligence. Future systems should combine physiology, biomechanics, environmental exposure, behavioural data and predictive analytics into continuous adaptive monitoring platforms. These systems should be judged by whether they improve context-specific decisions, not by the number of signals they collect. In sport, military, occupational and clinical environments, this may include identifying excessive heat strain, contextualising workload, supporting pacing or cooling decisions, or informing medical evaluation. However, these applications require different evidence thresholds. A system used for retrospective performance analysis does not require the same validation standard as one used to recommend athlete withdrawal or medical intervention.

The foundation for ecosystem-based monitoring is being laid through ongoing projects that combine validated measurement with AI-driven prediction. For example, at the upcoming National Games of China, we are expanding our work into sports such as cycling, where the interaction between environment, power output, and biomechanical efficiency can be modelled in real time. Drawing inspiration from Joyner’s 1991 analysis predicting the fastest possible marathon time [42], our goal is to develop an equivalent model for cycling—using individual athlete metrics, real-time environmental data, aerodynamic position, and road dynamics to predict optimal pacing and performance limits. This research remains ongoing and requires continued innovation. While the foundational work is complete, refinements are needed in miniaturisation, sensor comfort, and system reliability. We are, in many ways, at the “brick phone” stage of human performance sensing: the devices work, but they must evolve to become smaller, more durable, and more athlete-friendly.

At the Olympic and Paralympic Games in Paris 2024, the deployment of environmental monitoring systems directly at the field of play represented a technically significant step toward contextualised athlete monitoring in major sporting events. Athletes voluntarily wore validated biometric sensors during training and competition. Although these data were not used to guide operational decisions during the Games, such deployments illustrate the feasibility—and the current limitations—of sensor integration in live elite environments. International federations, including World Triathlon and World Sailing, have since expressed strong interest in adopting these technologies for future events, not only to monitor performance but also to safeguard health. These initiatives demonstrate that the integration of accurate sensing, AI interpretation, and environmental context is no longer hypothetical: it is actively unfolding, even if refinement and regulatory alignment remain ahead.

## 6. The Road Ahead: Scientific, Technical, and Systemic Challenges

Despite significant progress, substantial obstacles remain—scientific, systemic, and technological. The technical challenges are especially acute: ensuring long battery life, data reliability, safety, and adherence to data protection standards, while keeping devices lightweight and unobtrusive [4,26,43]. Regulatory acceptance remains a major barrier. In many sports, visible wearable devices, real-time feedback systems or external communication tools may be restricted during competition [44]. Consequently, future sensor ecosystems should be designed not only for technical validity, but also for sport-specific regulatory compatibility, including unobtrusive form factors, delayed-feedback modes or approved medical-monitoring pathways.

Equally critical is the need for interdisciplinary and cross-company collaboration. The current industry structure, where each company builds a proprietary sensor in isolation, is fundamentally at odds with scientific needs. Creating a truly integrated system that can measure, communicate, and interpret multi-sensor data demands coordination that few commercial entities can achieve alone. This has been the defining challenge and experience of our own projects: while the scientific rationale is strong, its implementation remains unresolved. Whether such ecosystems can become economically viable remains to be seen; for now, the research imperative drives progress. Of note, we do not attempt a formal feasibility or cost–benefit analysis here, as this is necessarily system- and context-specific and is a priority for future work. Qualitatively, the principal costs—hardware, integration, validation and governance overhead—should be weighed against the value of the specific decision being supported: where preventing a single exertional heat illness collapse or an inappropriate withdrawal carries high clinical and reputational value, a greater validation burden is justified, whereas low-stakes trend monitoring does not warrant the same investment.

Data governance is another critical challenge. Real-time physiological monitoring generates sensitive, high-frequency data that may reveal health status, fatigue, illness risk, tactical behaviour or performance capacity. Therefore, sensor ecosystems should define who owns the data, who can access it, how long it is stored for, whether it can be used for secondary purposes, and how athletes can provide informed consent in competitive environments. These issues become even more complex when AI-assisted systems generate risk scores or recommendations that may influence selection, withdrawal, return-to-play or medical decisions.

Continuous sensor ecosystems also introduce risks that extend beyond conventional data protection [43,45]. Poorly calibrated systems may create false reassurance, whereas overly sensitive systems may generate excessive false alarms and alert fatigue. Models trained on narrow samples may encode physiological, sex-based, ethnic, disability-related or sport-specific biases, leading to poorer performance in under-represented groups [41]. In elite sport, monitoring may also become coercive if athletes feel unable to refuse data collection because selection, contracts or medical clearance depend on participation [43]. Additional risks include athlete surveillance, asymmetric access to data by teams or commercial providers, opaque proprietary models, secondary use of physiological data for employment or insurance decisions, and commercial monopolisation of athlete data infrastructures [41,43]. These concerns do not argue against sensor ecosystems, but they require enforceable safeguards: purpose limitation, data minimisation, athlete access rights, independent audit, transparent model documentation, human oversight and clear separation between performance optimisation, medical monitoring and commercial exploitation. For AI-assisted components specifically, these safeguards should be operationalised as: model explainability appropriate to the decision (interpretable outputs or post hoc explanations that a clinician can scrutinise); continuous drift monitoring with scheduled recalibration; documented human oversight with a named accountable decision-maker; a defined regulatory-approval pathway where outputs inform medical decisions (for example, software-as-a-medical-device considerations); and post-deployment surveillance that tracks real-world performance, adverse events and false-alarm burden over time.

Notably, success depends on adhering to rigorous design and validation standards. Before any human sensing system is used to inform clinical or sporting decisions, it should satisfy a comprehensive set of requirements spanning analytical accuracy, safety, reliability, data integrity, interoperability, usability, durability, regulatory compliance and ethical governance.

## 7. Implementation Barriers and Strategies for Adoption

Beyond the scientific and technical obstacles above, translating physiological sensor ecosystems into practice faces human, organisational and financial barriers. Implementation-science frameworks show that health technologies frequently fail not because they do not work, but because these barriers are underestimated [46].

Human barriers: Adoption depends on the trust and workflow of those who must act on the outputs. Clinicians may distrust opaque or poorly validated alerts; athletes and workers may resist monitoring perceived as surveillance or as a threat to selection or employment [43,45]; and alert fatigue can erode responsiveness. Strategies include involving end-users in system design, providing interpretable outputs with explicit uncertainty [41], training staff in the meaning and limits of each metric, keeping an accountable human decision-maker for high-stakes actions, and making participation genuinely voluntary with transparent data-use terms.

Organisational barriers: Fragmented, proprietary systems and the absence of shared standards impede integration across teams, vendors and institutions [26,41], and responsibilities for data, alerts and decisions are frequently undefined. Strategies include adopting open data formats and interoperability standards [26], defining clear governance roles (data ownership, access, escalation pathways), embedding monitoring within existing medical and performance workflows rather than alongside them, and agreeing in advance how alerts translate into decisions.

Financial barriers: Hardware, integration, validation, data infrastructure and maintenance carry substantial and often recurring costs, while the return on investment is hard to quantify and reimbursement or funding pathways are typically absent. Strategies include prioritising investment according to the value of the specific decision supported, phased or shared deployment across events and institutions, cost-sharing through federated infrastructure, and building the health–economic and outcome evidence needed to justify adoption to funders, federations and regulators [4].

## 8. Conclusions

The decisive shift in human performance monitoring will not come from adding sensors, but from proving that integrated systems can be trusted to inform a specific decision. We have argued for three concrete moves: anchoring validation to the decision rather than the device (Table 2), attaching testable performance thresholds to each validation level, and embedding governance from the outset rather than retrofitting it. The near-term test cases are already defined—heat-strain management at World Triathlon and World Sailing events, and real-time pacing models at the 2025 National Games of China—and each will succeed or fail on validation and trust, not on data volume. We remain at the “brick-phone” stage of human sensing; the task for the coming years is not to sense more, but to sense in ways that are validated, interoperable, governed and decision-ready. The signal that matters is not the one we can measure, but the one we can act on safely.

## Figures and Tables

**Table 1:** Comparison of the proposed physiological sensor ecosystem with related paradigms, contrasting focus, validation emphasis, decision-support role and the gap each leaves for high-stakes sport and extreme-environment use.

Paradigm	Primary Focus	Validation Emphasis	Decision-Support Role	Gap for High-Stakes/Extreme-Environment Use
Digital-health ecosystems	Patient-centred health-data integration and care	Clinical/regulatory device validation	Clinical decision support	Not designed for competition constraints, field-of-play context or in-competition feedback limits
Cyber–physical (health) systems	Coupled sensing–computation–actuation loops	System reliability, latency, safety	Automated/closed-loop control	Limited treatment of athlete autonomy, governance and decision-specific evidence
Athlete-management systems	Aggregating training load, wellness, GPS/HR	Often single-variable device validation	Workload/readiness monitoring	Rarely anchored to a high-stakes medical decision or multi-level validation
Digital-twin approaches	Individualised simulation and counterfactuals	Model fidelity; data sufficiency	Predictive/what-if support	Data- and validation-intensive; immature for real-time field use
Multimodal-biosensing frameworks	Combining biosignals and biomarkers	Sensor-level analytical validity	Signal interpretation	Focus on measurement, not decision-anchored validation or governance
Proposed physiological sensor ecosystem (our proposal)	Decision-anchored integration for sport and extreme environments	Hierarchical, decision-specific thresholds (Table 2)	Fit-for-decision support (pacing to withdrawal) with human oversight	Integrates validation, governance and regulatory compatibility for a named decision

Note. The proposed ecosystem (in grey background) is distinguished by anchoring both validation and governance to a named high-stakes decision rather than to device measurement alone.

**Table 2:** Proposed, decision-specific validation thresholds for each level of the proposed framework.

Level	Validation Question	Proposed Minimum Threshold	Decision Tier
1. Analytical	Does the sensor capture the physical signal under controlled conditions?	Resolution ≤ 0.1 °C; bench drift ≤ 0.1 °C per hour; documented calibration	Prerequisite
2. Criterion	Does it agree with a reference standard in the intended context?	Systematic bias < 0.1 °C and 95% LoAs within ±0.4 °C vs. ingestible thermistor during exercise in the heat [13]	Screening
3. Reliability/sensitivity	Can it detect meaningful within-individual change over time?	Smallest detectable change < physiologically relevant change (≈0.3 °C); reported test–retest reliability	Trend monitoring
4. Ecological	Does it remain valid in real-world field conditions?	<10% data loss; limits of agreement maintained under motion, sweat and transmission constraints	Operational (event)
5. Decision (AI)	Do models or algorithms improve the health or performance decision?	Sensitivity ≥ 90% for EHI with an a priori-defined acceptable false-alarm burden; reported calibration	Withdrawal/medical intervention

Note. Thresholds are evidence-informed, decision-specific reference points, not universal cut-offs; they require prospective validation and context-specific adaptation (by decision, population, body site, activity and environment). Criterion-validity agreement limits are adapted from established body-core thermometry criteria [13,14]. °C, degrees Celsius; LoAs, limits of agreement; EHI, exertional heat illness.

**Table 3:** Mapping of artificial-intelligence model classes to representative use cases in real-time human performance monitoring.

Model Class	Example Use Case	When Preferred (Including Uncertainty-Aware Use)
Supervised learning	Classify risk states; estimate core-temperature trajectory or cardiovascular drift	Large labelled datasets; point predictions where calibrated probabilities suffice
Probabilistic/Bayesian	Update individual risk as new data arrive	Preferred when uncertainty is decision-relevant (e.g., withdrawal); yields calibrated uncertainty for high-stakes calls
Time-series (state-space, RNN, TCN, transformer)	Capture lagged workload–environment–physiology relationships	Strong temporal dependencies and delayed responses; can be made uncertainty-aware via probabilistic variants
Digital twins	Counterfactual simulation of pacing, cooling or exposure	Rich individual data available; pre-operational what-if analysis rather than real-time alerts
Edge–AI	Low-latency local inference	Latency- or privacy-critical settings; limited connectivity at the field of play
Federated learning	Multi-site model development without centralising data	Privacy and governance constraints across teams, events or institutions

Note. Uncertainty-aware (probabilistic or Bayesian) approaches are preferable wherever a recommendation can trigger a high-stakes action, or where data are sparse or noisy.

## Data Availability

No new data were created or analyzed in this study. Data sharing is not applicable to this article.

## References

[1] Muniz-Pardos B., Angeloudis K., Guppy F.M., Keramitsoglou I., Sutehall S., Bosch A., Tanisawa K., Hosokawa Y., Ash G.I., Schobersberger W. (2021). Wearable and Telemedicine Innovations for Olympic Events and Elite Sport. J. Sports Med. Phys. Fit..

[2] Guppy F., Muniz-Pardos B., Angeloudis K., Grivas G.V., Pitsiladis A., Bundy R., Zelenkova I., Tanisawa K., Akiyama H., Keramitsoglou I. (2023). Technology Innovation and Guardrails in Elite Sport: The Future Is Now. Sports Med..

[3] Düking P., Fuss F.K., Holmberg H.C., Sperlich B. (2018). Recommendations for Assessment of the Reliability, Sensitivity and Validity of Data Provided by Wearable Sensors. JMIR Mhealth Uhealth.

[4] Doherty C., Baldwin M., Keogh A., Caulfield B., Argent R. (2024). Keeping Pace with Wearables: A Living Umbrella Review of Systematic Reviews Evaluating the Accuracy of Consumer Wearable Technologies in Health Measurement. Sports Med..

[5] Gamage P.J., Fortington L.V., Finch C.F. (2020). Epidemiology of Exertional Heat Illnesses in Organised Sports: A Systematic Review. J. Sci. Med. Sport.

[6] Hollander K., Klöwer M., Richardson A., Navarro L., Racinais S., Scheer V., Murray A., Branco P., Timpka T., Junge A. (2021). Apparent Temperature and Heat-Related Illnesses during International Athletic Championships: A Prospective Cohort Study. Scand. J. Med. Sci. Sports.

[7] Racinais S., Hosokawa Y., Akama T., Bermon S., Bigard X., Casa D.J., Grundstein A., Jay O., Massey A., Migliorini S. (2023). IOC Consensus Statement on Recommendations and Regulations for Sport Events in the Heat. Br. J. Sports Med..

[8] Bandiera D., Racinais S., Garrandes F., Adami P.E., Bermon S., Pitsiladis Y.P., Tessitore A. (2024). Heat-Related Risk at Paris 2024: A Proposal for Classification and Review of International Federations Policies. Br. J. Sports Med..

[9] Vanos J.K., Grundstein A.J. (2020). Variations in Athlete Heat-Loss Potential Between Hot-Dry and Warm-Humid Environments at Equivalent Wet-Bulb Globe Temperature Thresholds. J. Athl. Train..

[10] Collins A., Brown M., Gutter B., Fuhrmann C. (2024). Microclimatic Variability and Thermal Comfort of Spectators in an Outdoor Stadium Venue. Atmosphere.

[11] Havenga H., Gharbi D., Sewry N., Language B., Neumann F.H., Finch J.M., Hill T., Boulter J., Jordaan E., Piketh S.J. (2024). Healthy Environments for AthleTes (HEAT): Environmental Conditions along a 90 Km Ultra-Marathon Event, South Africa. Int. J. Biometeorol..

[12] Bassett D.R., Rowlands A., Trost S.G. (2012). Calibration and Validation of Wearable Monitors. Med. Sci. Sports Exerc..

[13] Byrne C., Lim C.L. (2007). The Ingestible Telemetric Body Core Temperature Sensor: A Review of Validity and Exercise Applications. Br. J. Sports Med..

[14] Verdel N., Podlogar T., Ciuha U., Holmberg H.C., Debevec T., Supej M. (2021). Reliability and Validity of the CORE Sensor to Assess Core Body Temperature during Cycling Exercise. Sensors.

[15] Kaltsatou A., Anifanti M., Flouris A.D., Xiromerisiou G., Kouidi E. (2024). Validity of the CALERA Research Sensor to Assess Body Core Temperature during Maximum Exercise in Patients with Heart Failure. Sensors.

[16] McLaughlin B.C., Aguilera J.T., D’Lugos A.C. (2025). Validity of the CORE Wearable Sensor during Constant-Load Cycling Exercise in the Heat. J. Therm. Biol..

[17] Bongers C.C.W.G., Hopman M.T.E., Eijsvogels T.M.H. (2015). Using an Ingestible Telemetric Temperature Pill to Assess Gastrointestinal Temperature During Exercise. J. Vis. Exp..

[18] Mougin L., Cable T.G., Mears S.A., James L.J. (2025). Gastrointestinal Temperature Measurement from Ingestible Pills Provided 3 Hours Preexercise Is Insufficient to Avoid Interference Caused by Tepid Water Ingestion. Int. J. Sports Physiol. Perform..

[19] Domitrovich J.W., Cuddy J.S., Ruby B.C. (2010). Core-Temperature Sensor Ingestion Timing and Measurement Variability. J. Athl. Train..

[20] Grivas G.V., Muniz-Pardos B., Guppy F., Pitsiladis A., Bundy R., Miller M., Fitzpatrick D., Richardson A., Hodgson L., Leckie T. (2024). Assessing Core Body Temperature in a Cool Marathon Using Two Pill Ingestion Strategies. Trans. Exerc. Biomed..

[21] James C., Muniz-Pardos B., Ihsan M., Lo K.-K., Peña-Iglesias D., Angeloudis K., Teng Y., Jiao J., Hu K., Wong K. (2025). Similar Peak Core Temperatures in Amateur, Elite and World Cup Athletes During a World Cup Sprint Triathlon in the Heat. Sports Med..

[22] Esh C.J., Pitsiladis Y., Racinais S., Taylor L., Dablainville V., Belfekih T., Bendimerad F., Pitsiladis A., Verdoukas P., Willems M. (2025). Real-Time Monitoring of Biometric Responses During a 200-Km Ultra-Endurance Race Across the Desert. Eur. J. Sport Sci..

[23] Sakamoto Y., Alhadad S.B., Zhang X., Tan B.Y., Ang W.H., Law L.Y.L., Prakaash S., Racinais S., Muniz-Pardos B., Bandiera D. (2026). Physiological Demands and Contributors to Variance of Core Temperature in 162 Recreational Runners during Distance Running in a Warm-Humid Environment. Med. Sci. Sports Exerc..

[24] Muniz-Pardos B., Verdoukas P., Comadran de Barnola E., Chan-Twist Y.C.I., Al Tunaiji H., Pitsiladis Y. (2026). Real-Time Thermoregulatory and Cardiovascular Monitoring of Non- Acclimatised Mountaineers in Extreme Cold: A 10-Day Field Expedition Study. Front. Physiol..

[25] Byrom B., Watson C., Doll H., Coons S.J., Eremenco S., Ballinger R., Mc Carthy M., Crescioni M., O’Donohoe P., Howry C. (2018). Selection of and Evidentiary Considerations for Wearable Devices and Their Measurements for Use in Regulatory Decision Making: Recommendations from the EPRO Consortium. Value Health.

[26] Ash G.I., Stults-Kolehmainen M., Busa M.A., Gregory R., Garber C.E., Liu J., Gerstein M., Casajus J.A., Gonzalez-Aguero A., Constantinou D. (2020). Establishing a Global Standard for Wearable Devices in Sport and Fitness: Perspectives from the New England Chapter of the American College of Sports Medicine Members. Curr. Sports Med. Rep..

[27] Moyen N.E., Bapat R.C., Tan B., Hunt L.A., Jay O., Mündel T. (2021). Accuracy of Algorithm to Non-Invasively Predict Core Body Temperature Using the Kenzen Wearable Device. Int. J. Environ. Res. Public Health.

[28] Zhou D., Keogh J.W.L., Ma Y., Tong R.K.Y., Khan A.R., Jennings N.R. (2025). Artificial Intelligence in Sport: A Narrative Review of Applications, Challenges and Future Trends. J. Sports Sci..

[29] Seoni S., Jahmunah V., Salvi M., Barua P.D., Molinari F., Acharya U.R. (2023). Application of Uncertainty Quantification to Artificial Intelligence in Healthcare: A Review of Last Decade (2013–2023). Comput. Biol. Med..

[30] Mao S., Sejdic E. (2023). A Review of Recurrent Neural Network-Based Methods in Computational Physiology. IEEE Trans. Neural Netw. Learn. Syst..

[31] Katsoulakis E., Wang Q., Wu H., Shahriyari L., Fletcher R., Liu J., Achenie L., Liu H., Jackson P., Xiao Y. (2024). Digital Twins for Health: A Scoping Review. npj Digit. Med..

[32] Xi L., Li C., Anari M.S., Rezaee K. (2025). Integrating Wearable Health Devices with AI and Edge Computing for Personalized Rehabilitation. J. Cloud Comput..

[33] Wu S., Chatzimisios P., Daneshmand M., Khan H., Kavati R., Pulkaram S.S., Jalooli A. (2025). End-to-End Privacy-Aware Federated Learning for Wearable Health Devices via Encrypted Aggregation in Programmable Networks. Sensors.

[34] Killoughery I.T., Pitsiladis Y.P. (2024). Olympic AI Agenda: We Need Collaboration to Achieve Evolution. Br. J. Sports Med..

[35] Muniz-Pardos B., Sutehall S., Angeloudis K., Shurlock J., Pitsiladis Y.P. (2019). The Use of Technology to Protect the Health of Athletes During Sporting Competitions in the Heat. Front. Sports Act. Living.

[36] Buller M.J., Delves S.K., Fogarty A.L., Veenstra B.J. (2021). On the Real-Time Prevention and Monitoring of Exertional Heat Illness in Military Personnel. J. Sci. Med. Sport.

[37] Seshadri D.R., Li R.T., Voos J.E., Rowbottom J.R., Alfes C.M., Zorman C.A., Drummond C.K. (2019). Wearable Sensors for Monitoring the Physiological and Biochemical Profile of the Athlete. npj Digit. Med..

[38] Ramesh N., Joseph B. (2025). The Future of Workplaces: Monitoring Health and Safety Conditions Using Wearable Technologies. Indian J. Occup. Environ. Med..

[39] Theodoropoulos J.S., Bettle J., Kosy J.D. (2020). The Use of GPS and Inertial Devices for Player Monitoring in Team Sports: A Review of Current and Future Applications. Orthop. Rev..

[40] Peake J.M., Kerr G., Sullivan J.P. (2018). A Critical Review of Consumer Wearables, Mobile Applications, and Equipment for Providing Biofeedback, Monitoring Stress, and Sleep in Physically Active Populations. Front. Physiol..

[41] Canali S., Schiaffonati V., Aliverti A. (2022). Challenges and Recommendations for Wearable Devices in Digital Health: Data Quality, Interoperability, Health Equity, Fairness. PLoS Digit. Health.

[42] Joyner M.J. (1991). Modeling: Optimal Marathon Performance on the Basis of Physiological Factors. J. Appl. Physiol..

[43] Karkazis K., Fishman J.R. (2017). Tracking U.S. Professional Athletes: The Ethics of Biometric Technologies. Am. J. Bioeth..

[44] World Athletics Technical Rules. https://worldathletics.org/about-iaaf/documents/book-of-rules.

[45] Arnold J.F., Sade R.M. (2017). Wearable Technologies in Collegiate Sports: The Ethics of Collecting Biometric Data from Student-Athletes. Am. J. Bioeth..

[46] Greenhalgh T., Wherton J., Papoutsi C., Lynch J., Hughes G., A’Court C., Hinder S., Fahy N., Procter R., Shaw S. (2017). Beyond Adoption: A New Framework for Theorizing and Evaluating Nonadoption, Abandonment, and Challenges to the Scale-Up, Spread, and Sustainability of Health and Care Technologies. J. Med. Internet Res..

